# Health-related quality of life in a trial of acupuncture, sham acupuncture and conventional treatment for chronic sinusitis

**DOI:** 10.1186/1756-0500-1-37

**Published:** 2008-06-27

**Authors:** Knut Stavem, Edna Røssberg, Pål G Larsson

**Affiliations:** 1 Department of Pulmonary Medicine, Medical Division, Akershus University Hospital, Lørenskog, Norway; 2 Helse-Sør-Øst Health Services Research Centre, Research Centre, Lørenskog, Norway; 3 Faculty of Medicine, University of Oslo, Oslo, Norway; 4 Balder-klinikken, Oslo, Norway; 5 Heggeli helhetsmedisin, Oslo, Norway; 6 Department of Clinical Neurophysiology, The National Centre for Epilepsy, Division for Clinical Neuroscience, Rikshospitalet University Hospital, Oslo, Norway

## Abstract

**Background:**

Acupuncture is commonly used to treat chronic sinusitis, though there is little documentation on the effect. This study presents the health-related quality of life (HRQoL) outcomes in a trial comparing traditional Chinese acupuncture, sham acupuncture, and conventional treatment for chronic sinusitis.

**Findings:**

In a three-armed single blind randomized controlled study, we recruited 65 patients with symptoms of sinusitis >3 months and signs of sinusitis on computed tomography (CT). Patients were randomized to one of three study arms: (1) 2–4 weeks of medication with antibiotics, corticosteroids, 0.9% sodium chloride solution, and local decongestants (n = 21), (2) ten treatments with traditional Chinese acupuncture (n = 25), or (3) ten treatments with minimal acupuncture at non-acupoints (n = 19). Change in HRQoL was assessed over 12 weeks using the Chronic Sinusitis Survey (CSS) and Short form 36 (SF-36) questionnaires.

In the study, we found only a non-significant difference on the CSS symptom scale between conventional medical therapy and traditional Chinese acupuncture. On the SF-36 scale role-physical the change was larger in the conventional group than in the sham group (p = 0.02), and on the mental health scale the change in the conventional therapy arm was larger than in the traditional Chinese acupuncture group (p = 0.03). There was no difference in effect on HRQoL on any scale between the sham and traditional Chinese acupuncture groups.

**Conclusion:**

There was no clear evidence of the superiority of one treatment over another on short-term HRQoL outcomes, although there was a statistically non-significant advantage of conventional therapy in a few dimensions.

## Background

Treatment of chronic sinusitis is a well-established procedure in traditional Chinese acupuncture, and acupuncture is commonly used to relieve sinusitis and nasal symptoms [1-3]. The symptoms of chronic sinusitis are not easy to measure or quantify, and health-related quality of life (HRQoL) has recently gained increasing awareness as an outcome measure for interventions in chronic sinusitis [4-6].

Previous studies have reported a favourable effect of acupuncture in children and young adults with chronic maxillary sinusitis, compared with antibiotics and laser acupuncture [7], and that acupuncture is effective in sinusitis or sinus pain [8,9]. Recently, we reported only a nonsignificant advantage of conventional treatment compared with acupucture on symptoms in a three-armed randomized study for CT-verified chronic sinusitis [10]. The report focused on changes in symptoms, sinus soft tissue swelling on CT, and the two SF-36 component summary scales. However, in the study we also included a disease-specific outcome measure, the Chronic sinusitis survey (CSS), which was not validated in Norwegian at the time, but that might be more sensitive to change than the other HRQoL measures [4].

In the present study, we report the full HRQoL outcomes from this trial of conventional medical treatment, traditional Chinese acupuncture and minimal acupuncture at non-acupoints for CT-verified chronic sinusitis.

## Methods

### Subjects, study design and interventions

We included patients >17 years of age with sinusitis symptoms for >3 months and sinus swelling, fluid retention, or opacification on CT after screening >500 patients with sinusitis. We excluded patients if they were pregnant, had previously had acupuncture treatment, had been operated on for chronic sinusitis, had polypous sinusitis or pansinusitis, or used medication that could influence the results of the study. In total, 66 patients were included between 1996 and 2000. We recruited patients from one ear-nose-throat practice and advertised the study in local newspapers and a magazine. One otorhino-laryngologist examined and included all patients. He allocated them to one of three groups according to a six-block randomization algorithm, by first assigning a patient number to each patient. He then phoned one of the acupuncturists to receive information about the group allocation for that particular patient [10]. The groups were (1) conventional medical therapy, (2) traditional Chinese acupuncture (TCA), or (3) minimal acupuncture at non-acupoints (sham). No patients were included between February and September to avoid influence from seasonal allergies.

All patients in the conventional medical therapy group used xylometazoline as local vasoconstrictor, and 0.9% sodium chloride solution locally for one week, and oral corticosteroids for 14 days. In addition, 14 patients used cefalexin 1500 mg daily for 10 days and six used azithromycin 500 mg for 7 days.

Both acupuncture groups had 10 treatments with bilateral acupoints over 4 weeks, performed by the same experienced acupuncturists with 4–10 years of experience and with experience in the treatment of chronic sinusitis. The TCA group patients had individual TCM diagnoses and treatment using 1.0–1.5 cun needles (Ø 0.28 mm, length 25–40 mm). The needles were inserted to from 0.5 cun (facial/hand/feet area) to a maximum of 1.3 cun (arms/legs/trunk area) depth to achieve a good needle sensation, stimulated manually using reducing or reinforcing methods and left in the acupoints for 25 minutes [10,11].

In the sham treatment group, the patients were given minimal acupuncture at non-acupoints outside the meridians. One point was situated on each shoulder between LI 15 and TE 14, one on each thigh 3 cun above the midpoint of the patella, and two bilateral points were situated 2.5 cun lateral to the umbilicus. For the sham group, we used 0.5 cun needles (Ø 0.25 mm, length 13 mm) A shallow, superficial insertion of the needle (maximum depth 0.25 cun) and minimal needle sensation was emphasized. The needles were left in the points for 25 minutes[10,12]. We applied the same sham points during each treatment session. In all treatment groups, medication used for other indications remained unchanged.

### Outcome assessment

The participants responded to a questionnaire in the physician's office at baseline and after 12 weeks, including the Chronic sinusitis survey and Short Form 36 questionnaires.

#### Chronic sinusitis survey

The Chronic sinusitis survey (CSS) is a 6-item duration-based, sinus-specific questionnaire with a symptom and a medication subscale for use in chronic sinusitis [4,5,13]. Scores were reported on a 0–100 scale, where 100 represents minimal symtoms or medication use. The Norwegian version of the CSS has only recently been validated [14].

#### Short form 36

The Short form 36 (SF-36) assesses eight dimensions of health status including physical functioning, role limitations due to physical problems, bodily pain, general health, vitality, social functioning, role limitations due to emotional problems, and mental health [15,16]. The scales were scored from 0 (lowest level of functioning) to 100 (highest level of functioning). The SF-36 has been extensively validated [15,16] and used in subjects with chronic rhinosinusitis [13,17-19]. We used the Norwegian standard SF-36 version 1.2 [20].

### Statistical analysis

Descriptive statistics are presented with means and SDs, or percentages. We compared changes in HRQoL from baseline to 12 weeks between groups using one-way analysis of variance with post-hoc Tukey's test because of multiple comparisons.

The required sample size was initially estimated to two groups of 22 patients, to detect a group difference of 0.85 SD on a visual analog scale, with power 0.8 and a 5% confidence level. Before study start, the study protocol was adjusted to include a third arm with 22 patients [10].

We omitted pairwise comparisons of changes on the CSS medication effects and total scales involving the conventional treatment group, because medication was part of the protocol for this group.

We chose a 5% confidence level, using two-sided tests. The Regional Committee for Medical Research Ethics and The Norwegian Data Inspectorate approved the study.

## Results

The intervention groups were reasonably balanced (Table 1). On baseline sinus CT, three patients in the conventional medicine group had opacification, six in the sham group, and eight in the TCA group. Only two patients in the TCA group had baseline fluid retention.

**Table 1:** Patient baseline characteristics and HRQoL scores according to allocation group, mean (SD)

	**Acupuncture**	**Sham**	**Conventional**
n	23–25	17–19	19–21
Women, number (%)	11 (44)	9 (47)	13 (62)
Age	41 (14)	47 (14)	42 (13)
Duration of chronic sinusitis in years	7 (8)	12 (14)	10 (11)
*Chronic sinusitis survey*^1^			
Sinusitis symptoms	48 (23)	40 (24)	33 (31)
Medication effects	84 (21)	85 (16)	82 (15)
Total	65 (16)	62 (16)	58 (15)
*Short form 36*^2^			
Physical functioning	77 (25)	85 (15)	86 (15)
Role – physical	48 (41)	61 (40)	54 (41)
Bodily pain	57 (22)	61 (25)	60 (25)
General health	48 (21)	71 (17)	60 (25)
Vitality	42 (24)	51 (21)	49 (23)
Social functioning	67 (27)	72 (23)	69 (28)
Role – emotional	61 (38)	69 (34)	67 (42)
Mental health	75 (16)	78 (11)	69 (21)

^1 ^0 (maximal) to 100 (minimal symptoms/medication use) scale, ^2 ^0 (lowest) to-100 (highest level of functioning) scale

The completion rate at 12 weeks was 17/25 in the TCA group, 15/19 in the sham group, and 17/21 in the conventional medicine group. Completers, who responded to the sinusitis symptoms scale of the CSS at baseline and after 12 weeks (n = 47), were somewhat older, had had chronic sinusitis longer, and had better baseline CSS and SF-36 scores on all subscales than dropouts (n = 18) after 12 weeks, although only the differences on the SF-36 General health (p = 0.03) and Social functioning scales (p = 0.04) were statistically significant. From baseline to 12 weeks, there was a trend to improvement on the CSS sinusitis symptoms scale in all three allocation groups, but there was no statistical difference in the changes between groups (Table 2).

**Table 2:** Change in HRQoL scores from baseline to 12 weeks, mean (SD) unless otherwise stated

	**Change score**			**Mean difference between change scores**								
		
	**(1) Acupuncture**	**(2) Sham**	**(3) Conventional**	**(1) – (2)**	**(95%CI)**	**p**^3^	**(3) – (2)**	**(95%CI)**	**p**^3^	**(3) – (1)**	**(95%CI)**	**p**^3^
				
*n*	*15–18*	*14–16*	*16–17*									
*Chronic sinusitis survey*^1^												
Sinusitis symptoms	11 (23)	8 (24)	32 (41)	3	(-24 to 31)	0.96	24	(-3 to 51)	0.09	24	(-3 to 52)	0.14
Medication effects	-0.5 (18)	8 (22)	-10 (21)	-9	(-26 to 9)	0.44			*			*
Total	6 (15)	7 (18)	10 (24)	-1	(-19 to 17)	0.99			*			*
*Short Form 36*^2^												
Physical functioning	1 (16)	0.5 (13)	6 (17)	1	(-12 to 14)	0.99	5	(-8 to 18)	0.58	5	(-8 to 17)	0.68
Role – physical	-3 (48)	-12 (39)	28 (36)	9	(-44 to 25)	0.8	40	(5 to 75)	0.02	31	(-3 to 65)	0.08
Bodily pain	2 (23)	5 (23)	16 (20)	-3	(-21 to 15)	0.92	11	(-7 to 29)	0.33	14	(-4 to 32)	0.16
General health	2 (22)	-4 (18)	10 (21)	6	(-11 to 23)	0.67	14	(-3 to 31)	0.13	8	(-9 to 25)	0.48
Vitality	-1 (20)	4 (16)	13 (31)	-5	(-25 to 14)	0.8	8	(-11 to 28)	0.56	13	(-6 to 33)	0.21
Social functioning	7 (15)	5 (22)	15 (23)	1	(-15 to 18)	0.98	9	(-8 to 26)	0.4	8	(-9 to 24)	0.5
Role – emotional	-4 (36)	7 (31)	24 (40)	-10	(-41 to 20)	0.69	17	(-14 to 48)	0.4	27	(-2 to 57)	0.08
Mental health	-3 (10)	0 (12)	10 (20)	-3	(-15 to 9)	0.82	10	(-2 to 22)	0.12	13	(1 to 25)	0.03

Change = last value-first value; a negative change score represents reductions in symptoms; * omitted because of medication use in the conventional treatment arm; ^1 ^0 (maximal) to 100 (minimal symptoms/medication use) scale, ^2 ^0 (lowest) to-100 (highest level of functioning) scale; ^3 ^after adjustment for multiple comparisons

In the conventional treatment group all eight SF-36 scales improved over the 12-week period (Table 2). Only two comparisons of the improvements were statistically significant: conventional medicine vs. sham on the Role-physical scale and conventional medicine vs. TCA on the Mental health scale. There was no statistical difference between TCA and sham on any of the SF-36 scales (Table 2).

## Discussion

In this study of patients with CT-verified chronic sinusitis, there was a small improvement on the CSS sinusitis symptom scale in all three groups over 12 weeks. The improvement was largest for conventional treatment, however statistically not significantly different from the change in the two other allocation groups. On the eight SF-36 scales, there were only significant differences in favor of the conventional group on one scale compared with TCA and on one scale compared with sham.

The short-term changes in the present report are in line with our previous report using CT soft tissue swelling, symptoms and summary scales of the SF-36 as outcomes [10]. Comparison with other studies is difficult because of differences in criteria for establishing the diagnosis of sinusitis, patient selection, lack of feasible control groups, and use of different outcomes [7]. There was some difference in the proportion of patients with opacification on sinus CT between the randomized groups. In this randomized study, this distribution was by chance. We have no reason to believe that this influenced the outcome of the study. Further, we cannot exclude that some of the patients may have had symptoms of perennial rhinitis, though we have no reason to believe that this contributed to a difference in outcome between the three groups.

A strength of the present study is the randomized design and the blinding of the patients to the type of acupuncture given. We used validated instruments for HRQoL assessment; the disease-specific CSS and the well-known SF-36. The small sample size, and the three intervention groups, which required adjustment for multiple comparisons, limited the study power. It is possible that the study was underpowered, and that the differences in favor of the conventional treatment group would have been statistically significant in a larger study.

We used minimal acupuncture at non-acupoints to emulate TCA, using a penetrating or invasive sham procedure on points that were considered inappropriate for treatment of sinusitis. The lack of standardization of treatments may represent a limitation of the study. The TCA was not standardized, but tailored to the individual patient according to the traditional Chinese medicine diagnosis. Similarly, the conventional medical therapy was not entirely standardized, although all patients had a common core of local therapy and oral corticosteroids.

Because we included only patients with CT-verified chronic sinusitis, only 10–15% of the examined patients were eligible. CT-verified sinusitis is poorly associated with symptoms [21-23]. Therefore, one should be cautious with generalization beyond patients with CT-verified sinus soft tissue swelling. In clinical practice the treatment of sinusitis is initiated without CT scan, and the diagnosis of chronic sinusitis typically implies having had sinusitis symptoms for >3 months. Finally, our patients had longer duration of chronic sinusitis, with mean symptom duration >9 years, and therefore may represent a therapy-resistant population.

## Conclusion

We conclude that in this single blind randomized trial, there was no clear evidence of short-term improvement of one treatment over another. However, there was a non-significant advantage of conventional therapy, but this should be reassessed in a larger trial and with less restrictive inclusion criteria. There was no difference in change in HRQoL between TCA and minimal acupuncture at non-acupoints.

## Competing interests

The authors declare that they have no competing interests.

## Authors' contributions

KS, ER and PGL participated in the study design and prepared the protocol. ER organized the data collection and was one of the acupuncturists. KS and PGL cleaned the data and performed the data analysis. KS drafted and revised the paper. ER and PGL critically reviewed and commented on the paper. All authors approved the final version.
